# Antimicrobial potential, phytochemical profile, cytotoxic and genotoxic screening of *Sedum praealtum* A. DC. (balsam)

**DOI:** 10.1186/s12906-020-02915-6

**Published:** 2020-04-29

**Authors:** Marcelo Fabiano Gomes Boriollo, Milene Bueno Marques, Thaísla Andrielle da Silva, Jeferson Júnior da Silva, Reigson Alves Dias, Thyago Henrique Neves Silva Filho, Isadora Letícia Ribeiro Melo, Carlos Tadeu dos Santos Dias, Wagner Luís de Carvalho Bernardo, Nelma de Mello Silva Oliveira, Vera Maria Peters, José Francisco Höfling, Denise Madalena Palomari Spolidorio

**Affiliations:** 1 Laboratory of Microbiology and Immunology, Department of Oral Diagnosis, Dental School of Piracicaba, State University of Campinas (FOP/UNICAMP), Piracicaba, SP 13414-903 Brazil; 2 Center for Research and Postgraduate Studies in Animal Science, Pathology and Animal Pharmacology Area, University of Alfenas (UNIFENAS), Alfenas, MG 37132-440 Brazil; 3 Laboratory of Pharmacogenetics and Molecular Biology, Faculty of Medical Sciences, University of Alfenas (UNIFENAS), Alfenas, MG 37132-440 Brazil; 4 Department of Exact Sciences, College of Agriculture, University of São Paulo (ESALQ/USP), Piracicaba, SP 13418-900 Brazil; 5 Laboratory of Oral Microbiology and Immunology, Department of Physiology and Oral Pathology, Araraquara School of Dentistry, São Paulo State University (FOAr/UNESP), Araraquara, SP 14801-903 Brazil; 6 Reproductive Biology Center, Federal University of Juiz de Fora (UFJF), Juiz de Fora, MG 36036-900 Brazil

**Keywords:** Antimicrobial susceptibility, Cytotoxicity, Genotoxicity, Phytochemical screening, *Sedum praealtum* a. DC

## Abstract

**Background:**

*Sedum praealtum* has been used for a long time in traditional medicine as an analgesic and anti-inflammatory agent. Its beneficial effects have been known since ancient times, when Latinos used it to treat sore and swollen eyes. This research evaluated the antimicrobial potential, the cytotoxic and genotoxic effects, and some chromatographic profiles of the hydroethanolic extract of leaves, stems and roots of *S. praealtum*.

**Methods:**

The antimicrobial activities were carried out by broth microdilution and agar diffusion. In vitro cytotoxicity was evaluated by cell cultures of *Aedes albopictus* and the selectivity index (SI) was estimated: SI=CI_50_/MIC. Genotoxic and systemic toxic effects of *S. praealtum* leaves were analyzed by micronucleus assay in mice bone marrow. Chromatographic profiles and mass spectra were investigated by GC-MS.

**Results:**

Gram-positive (*B. subtilis*, *B. cereus*, *M. luteus*, *E. faecalis* and *S. aureus*) and gram-negative (*E. coli*, *E. aerogenes*, *S. marcescens*, *P. aeruginosa*, *P. mirabilis* and *S. typhimurium*) bacteria exhibited MICs ranging from 12.5–50 and 0–50 mg/ml, respectively. *Sedum praealtum* showed no efficacy against *M. tuberculosis* and *M. bovis*. Cytotoxicity (CI_50_) of *S. praealtum* was 4.22 and 5.96 mg/ml for leaves and stems, respectively, while its roots showed no cytotoxicity. Micronucleated polychromatic erythrocytes (MNPCEs) analyzes showed no differences between treatment doses (0.5–2 g/kg) and negative control (NaCl), but the PCE/NCE ratio (polychromatic erythrocyte/normochromatic erythrocyte) showed significant differences. Phytochemical screening identified thirteen compounds in the leaves, stems and roots of *S. praealtum* potentially associated with their biological activities.

**Conclusions:**

This research comprises a first scientific study on genotoxicity, cytotoxicity and antimicrobial effects of *S. praealtum* (Balsam), and it provides an initial theoretical foundation for its comprehensive use. Results showed antibacterial action of *S. praealtum* against gram-positive bacteria and some gram-negative species (depending on the plant anatomical part), but ineffective antimycobacterial action. However, *S. praealtum* leaves and stems display potential cytotoxicity, contributing to the SI < 1 values. In addition, *S. praealtum* leaves exhibit no clastogenic and/or aneugenic effects, but it has systemic toxicity dose-independent.

## Background

The genus *Sedum* (family Crassulaceae) features more than 350 species, which encompasses a large number of pharmacologically active species. Chemical studies of *Sedum* species have led to the isolation of several classes of substances, such as alkaloids, tannins, flavonoids and cyanogenic compounds [1–4]. The *Sedum praealtum* A. DC. species (synonym. *Sedum dendroideum* and *Sedum dendroideum ssp. praealtum* (A. DC.) [5–7] is a little bush with yellow flowers, found from Mexico to Guatemala, popularly known as Balsam [6]. According to classification, balm occupies the following taxonomic position: Division: *Magnoliophyta*; Class: *Magnoliopsida*; Subclass: *Rosidae*; Order: *Rosales*; Family: *Crassulaceae*; Genus: *Sedum*; Species: *Sedum dendroideum subsp. praealtum* (DC.) [8]. Balm is native to semidesert areas, 30 to 60 cm tall, fleshy, smooth, spatulated and recurved leaves gathered in whorls. Its inflorescences are terminal and branched, with numerous yellow flowers, formed in autumn and winter [9]. Its cultivation occurs singly or in groups, in full sun and fertile and permeable soil, as well as in rock gardens, being a drought- and frost-resistant plant. The juice of its leaves is said to be healing. The spread of Balsam (*Sedum praealtum*) is mainly due to the cutting of branches, which must be planted in 0.5 × 0.5 m pits when plants have five to eight definite leaves [8].

Its beneficial effects have been known since ancient times, when Latinos used it to treat sore [10] and swollen eyes [11]. An aqueous decoction of *S. praealtum* parts has been used to ulcer treatments [12, 13], general inflammatory problems [14] and as contraceptive [15]. A preliminary study of the water extract of *S. praealtum* – however, under the name of *S. dendroideum* (Crassulaceae) – showed an inhibitory effect on the motility of human sperm, as well as an anti-fertilization activity in Sprague-Dawley rats [16]. In another study, employing acetic acid-induced writhing (antinociceptive activity), croton oil-induced ear edema and carrageenan-induced peritonitis (anti-inflammatory activity) models in mice, it has been demonstrated that the main kaempferol glycosides can be responsible for the medicinal use of *S. dendroideum* against pain and inflammatory problems [13]. However, antimicrobial effects were reported only in two other species of the genus *Sedum*, *S. aizoon*, and *S. tatarinowii* [17]. The antimicrobial activity of medicinal plants has been attributed to small terpenoids and phenolics such as thymol, carvone, carvacrol, menthol and murolene, which also, in pure form, show antifungal or antibacterial activity. Although the mechanisms of action have been poorly characterized, this seems to be associated with the lipophilic nature of the compounds, with a buildup in membranes and loss of energy by the cells [18].

Biologically active compounds have been recognized for their pharmacological properties; however, several of these compounds could not be used in therapy because of their toxicological, carcinogenic, and mutagenic properties [19]. In the development of new drugs, analyses of genotoxicity assays represent a considerable weight, since most pharmaceutical industries deliberate the processing of new therapeutic agents also based on the data of in vitro and in vivo genotoxicity [20]. Thus, the assays for evaluation of mutagenic activity of plants used by the population as well as their isolated substances are essential to establish control measures on indiscriminate use. In addition, it is necessary to clarify the mechanisms and conditions that decided the biological effect, before the plants are viewed as therapeutic agents [21]. The in vivo micronucleus (MN) assay in mice bone marrow plays a crucial role in tests that aim to identify risks for mutagen agents [22], especially the evaluation of mutagenic risks that allows the consideration of in vivo metabolic factors, pharmacokinetics, and DNA repair processes although these may vary among species, tissues and genetic mechanisms [23]. In addition, the knowledge of genotoxic effects induced by herbal medicines and foods using the MN in vivo assay in mammals has been the aim of several research groups [24–27].

Although little information about the popular knowledge and the scientific research support the potential therapeutic effectiveness of the aqueous decoction, aqueous extract or lyophilization of balsam juice (*S. praealtum*), as ocular symptoms [10, 11], gastric ulcer [12, 13], anti-inflammatory action [14], contraceptive [15], inhibition of human sperm motility and anti-fertilization activity in mice [16], a limited and/or non-existent number of investigations aimed to examine the antimicrobial – only the *Sedum aizoon* and *Sedum tatarinowii* species [17] – and genotoxic effects has been observed so far. Aiming to contribute with information about the antimicrobial and genotoxic potential of herbal medicine, particularly of the hydroethanolic extract of the stem, leaf, and root of *S. praealtum* (Balsam), we evaluated (*i*) in vitro antibacterial, antimycobacterial and antifungal action employing broth microdilution and the agar diffusion test and (*ii*) the in vitro cytotoxic activity using cellular culture of *Aedes albopictus*. We also analyzed the genotoxic effects of *S. praealtum* leaves through the in vivo micronucleus assay in mice bone marrow.

## Methods

### Plant extract

The whole plant of *S. praealtum* (stem, leaf and root) was collected from the urban area of the Alfenas city, Minas Gerais State, Brazil (21° 24′ 44.98″ S and 45° 57′ 39.87″ W, elevation of 818 m). This plant has been kindly identified by a plant taxonomist (Federal University of Alfenas – UNIFAL-MG) and filed in the Rede Nacional de Herbários da Sociedade Botânica do Brasil (UNIFAL-MG UALF), voucher specimen no. 1024. Anatomical parts of the plant (stem, leaf and root) were cleaned and manually cut and hydroalcoholic extracts have been macerated (200 mg/ml) for 7 days in ethanol 70 GL, in the dark and under daily stirring. Then, these extracts were subjected to the filtering process using nylon and paper filters.

Prior to antimicrobial susceptibility testing, cytotoxicity and genotoxicity assays, aliquots (500 ml) of these extracts were submitted to solvent removal proceedings by rotary evaporation (40 rpm) (Rotary Evaporator RV 10 Control V, IKA® Works, Inc., USA) coupled in bath heating systems (40 °C) (Heating Baths HB10, IKA® Works, Inc., USA), vacuum pump (175 mbar) (Chemistry diaphragm pump MD 1C, VACUUBRAND GMBH + CO KG, Wertheim, Germany), recirculator of distilled water (10 °C) (Banho Ultratermostatizado Microprocessado Digital, SPLABOR, cod. # SP-152/10, Presidente Prudente, SP, Brazil) and evaporation bottle (RV 10.85 Evaporation Flask, NS 29/32-2 L, IKA® Works, Inc., USA). The final product was transferred to 1 L reaction bottle (SCHOTT® DURAN®) and kept at − 20 °C for 24 h, to evaluate the freezing of the final product and the efficacy of the solvent evaporation process [27]. Then, aliquots (15 ml) of this final product were transferred to penicillin glass vials (50 ml) and lyophilized (0.12 mbar at − 50 °C) (Lyophilizer model Alpha 1–2 LDPlus, Martin Christ Gefrier trocknung sanlagen GmbH©, Germany) and their dry mass was measured (Electronic Analytical Balance AUW-220D, Shimadzu Corp., Kyoto, Japan). The lyophilized final product (stem, leaf and root of *S. praealtum*) was prepared in aqueous solvent (Type 1 water, Sistema Milli-Q Direct 8, Millipore Indústria e Comércio Ltda., Barueri, SP, Brazil) at concentrations of 20× (in vitro antimicrobial susceptibility testing) and 2× (in vitro cytotoxicity and in vivo genotoxicity assays), sterilized by filtration (Millipore Corporation, hydrophilic Durapore® PVDF, 0.22 μm, ∅ 47 mm, cat. # GVWP 047 00), and stored in sterile polypropylene tubes (50 ml) at − 70 °C until use.

### Qualitative analysis by gas chromatography – mass spectrometry (GC-MS)

The lyophilized final product (stem, leaf and root of *S. praealtum*) was partially dissolved with ethanol. The dispersions were filtered and analyzed by GC-MS (Aligent 5975C TAD Series GC/MSD System,©Aligent Technologies, Inc., CA, USA) using the following chromatographic conditions: (*i*) Sample: injected volume of 2.0 μl; (*ii*) Column: HP-5MS, 5% diphenyl, 95% dimethylpolysiloxane (30 m × 0.25 mm × 0.25 μm); (*iii*) Drag gas: He (99.9999) → 1 ml/min; (*iv*) Injector: 280 °C, Split 1:10 (leaf and root) and 1:1 (stem); (*v*) Oven: 50 °C (2 min) → 250 °C (5 °C/1 min); 250 °C (10 min); and (*vi*) Detector: Linear quadrupole mass spectrometer, Ionization source (impact by electrons → 70 eV), Scan mode (0.5 s/scan), Mass range [33–500 Da (u)], Line transfer (280 °C) and Filament (off at 7.0 min). The Mass Spectral Database (NIST 11) was used to identify compounds detected in the chromatograms [28].

### Microbial strains

A total of 11 bacterial strains (*Bacillus subtilis* ATCC® 6633, *Bacillus cereus* ATCC® 11778, *Micrococcus luteus* ATCC® 9341, *Enterococcus faecalis* ATCC® 51299, *Staphylococcus aureus* ATCC® 6538, *Escherichia coli* ATCC® 25922, *Serratia marcescens* LMI-UNIFAL, *Pseudomonas aeruginosa* ATCC® 27853, *Proteus mirabilis* ATCC® 25922, *Salmonella typhimurium* ATCC® 14028 and *Enterobacter cloacae* LMI-UNIFAL), 2 strains of yeasts (*Candida albicans* ATCC® 10231 and *Saccharomyces cerevisiae* ATCC® 2601) and 2 mycobacterial strains (*Mycobacterium tuberculosis* ATCC® 25177 [H37Ra] and *Mycobacterium bovis* [BCG]), belonging to the bacteria collection of the Laboratory of Pharmacogenetics and Molecular Biology, University of Alfenas (UNIFENAS), were subjected to susceptibility tests against lyophilized extracts of *S. praealtum*.

### Agar diffusion method (bacteria and yeasts)

The profile of the in vitro antimicrobial susceptibility of bacterial and yeast strains against *S. praealtum* extracts was determined by the agar diffusion method, following the guidelines established by the Clinical and Laboratory Standards Institute [29–31], with some adaptations [32]. Prior to testing, bacteria and yeasts were grown in BHI agar at 35 °C for 24 h and in SDA agar at 35 °C for 24 h, respectively. Then, a 10-μl loop inoculum of each microbial sample was resuspended in 5 ml of sterile saline solution (145 mM NaCl) and set to a turbidity of 0.5 on the McFarland scale (bacteria: 1–4 × 10^8^ CFU/ml; yeasts: 1–5 × 10^6^ CFU/ml) or equivalent to a transmittance of 79.5–83.2% using spectrophotometer (Thermo Scientific Multiskan GO UV/Vis, Microplate and Cuvette Spectrophotometer, ref. # 51119200, Thermo Fisher Scientific Inc., Waltham, MA, USA) with 625 nm wavelength (bacteria) and 530 nm (yeasts) (T = 79.5–83.2% → A _625 nm/530 nm_ = 2 – log_10_%T → A _625 nm/530 nm_ = 0.100–0.080). This cell suspension was vortex-stirred for 15 s and plated (Spread-Plate method) on sterile MH agar medium, 7.2 to 7.4 pH (Mueller Hinton no. 2 Control Cations, code # M1657, Himedia), and on sterile MH agar medium supplemented with 2% glucose for bacteria and yeasts, respectively, previously prepared in Petri dish (150 × 15 mm; 50 ml growth medium/plate; height in each plate equal to 4 ± 0.5 mm). These plates were kept at room temperature for 15 min to complete absorption of the inoculum into the culture medium. Then, 40 μl of each extract (100 mg/ml) were poured in wells (4 mm) evenly made on the surface of the inoculated culture medium, and the plates were inversely incubated at 35 °C for 24 h. These tests were performed in triplicate assay systems and the interpretation of results was carried out from the zone of inhibition of microbial growth (∅ of the halo in mm). Chlorhexidine to 0.12% (m/v) and Type 1 water were used as positive control (zone of inhibition) and negative (lack of zone of inhibition), respectively.

### Microdilution method

The minimum inhibitory concentration (MIC) of *S. praealtum* extracts against bacterial and yeast strains was determined by the broth microdilution method, following the guidelines established by the Clinical and Laboratory Standards Institute [33–35], with some adaptations [32]. The concentrations tested covered a range of 50, 25, 12.5, 6.25, 3.125, 1.562, 0.781, 0.390, 0.195, 0,097, 0.048 and 0.024 mg/ml. These tests were performed in triplicate assay systems employing microdilution plates with multiple wells (96-well cell culture microplates, flat-bottom, Corning Inc., NY, USA), containing 50 μl of sterile MH agar medium, 7.2 to 7.4 pH for bacteria, and sterile MH agar medium supplemented with 2% glucose for yeasts, in ultrapure Type 1 water.

Prior to the tests:

(*i*) standard solutions of lyophilized extracts (concentrations of 20×) were diluted in MH liquid culture medium. Thus, 50 μl aliquots of these dilutions were applied to the first wells of each row of the microdilution plates containing MH liquid culture medium (50 μl), according to the procedures of serial dilutions (2×), to create different concentrations of the extracts to be tested;

(*ii*) bacteria and yeasts were grown in BHI and SDA agar, respectively, at 35 °C for 24 h. Then, a 10-μl loop inoculum of each microbial sample was resuspended in 5 ml of sterile saline solution (145 mM NaCl) and set to a turbidity of 0.5 on the McFarland scale (bacteria: 1 × 10^8^ CFU/ml; yeasts: 1–5 × 10^6^ CFU/ml) or equivalent to a transmittance of 79.5–83.2% using a spectrophotometer with a wavelength of 625 nm (bacteria) and 530 nm (yeasts) (T = 79.5–83.2% → A _625 nm/530 nm_ = 2 – log_10_%T → A _625 nm/530 nm_ = 0.100–0.080). The bacterial cell suspension was vortex-stirred for 15 s and diluted in a 1:10 proportion in sterile saline solution (1 × 10^7^ CFU/ml). In turn, the yeast cell suspension was vortex-stirred for 15 s and diluted in a 1:10 proportion in sterile saline solution (1–5 × 10^5^ CFU/ml), followed by a new 1:10 dilution (1–5 × 10^4^ CFU/ml). During the assays, aliquots of 5 μl of each working inoculum (5% of well volume) were placed in the microdilution wells, containing 100 μl/well of MH liquid culture medium and different concentrations of the products to be tested (*S. praealtum*): final concentration of the bacterial inoculum equal to 5 × 10^5^ CFU/ml and yeast inoculum equal to 0.5–2.5 × 10^3^ CFU/ml.

Afterwards, these microdilution plates were incubated at 35 °C for 24 h (bacteria) and 48 h (yeasts). Soon after the incubation period, 30 μl aliquots of resazurin solution (Resazurin sodium salt, Cat. #R7017, Sigma-Aldrich Co., St. Louis, MO., USA), previously prepared in 0.02% (m/v) in Type 1 water, sterilized by filtration and stocked at 4 °C for up to 1 week, were added to each well and the plates were reincubated overnight. Interpretation of results was made through visual reading of test plates. A change in color from blue to pink indicated microbial growth. MIC was defined as the lowest concentration of drug that prevented this color change [36].

### Minimum microbicidal concentration (MMC).

The minimum microbicide concentration (MMC) was determined according to the modifications previously proposed [37]. For each microbial strain, 50 μl aliquots of the total volume of the well corresponding to the MIC were homogenized with a pipette and grown in Petri dishes (90 × 15 mm) containing BHI and SDA agar to bacteria and yeasts, respectively. Each aliquot was placed gently at a given point on the growing medium and kept at room temperature for complete absorption. Therefore, the plate was streaked to separate microorganisms and remove them from drug sources (*S. praealtum* extracts) [38], and incubated at 35 °C for 46–48 h. MMC was the lowest concentration of the drug (extract) able to eliminate ≥99.9% of the final inoculum (0.05–0.25 colony), whereas microstatic activity was defined from the reduction of ≤99.9% of the final inoculum [37].

### Agar diffusion method (mycobacteria)

The profile of the antimicrobial susceptibility against *S. praealtum* extracts was determined by the agar diffusion method, following the guidelines established by the Clinical and Laboratory Standards Institute [39]. *M. tuberculosis* ATCC® 25,177 (H37Ra) and *M. bovis* (BCG) strains were tested on Middlebrook 7H10 agar (Middlebrook 7H10 AgarBD, Cat. #295964, Becton, Dickinson and Company, New Jersey, USA), with Middlebrook OADC Enrichment added (BBL Middlebrook OADC Enrichment, Cat. #212240, Becton, Dickinson and Company, New Jersey, USA). Prior to testing, filter paper discs (10 mm ∅) were moistened with solutions of extracts (50 mg/ml) and oven-dried at 37 °C. Discs containing 0.12% m/v chlorhexidine and Type 1 water were used as positive control (zone of inhibition) and negative (lack of zone of inhibition), respectively. Freshly cultivated mycobacterial cells were resuspended in 5 ml of sterile saline solution (145 mM NaCl), adjusted to a turbidity of 0.5 on the McFarland scale, with the aid of glass beads for breakdown of the colonies, and poured (Spread-Plate method) on the agar. Agars were kept at room temperature for 15 min to complete absorption of the inoculum. Afterwards, discs containing *S. praealtum* extracts were dispensed on the surface of the inoculated culture medium, and the plates were inversely incubated at 35 °C for 8 weeks (i.e., cultures were analyzed within 5–7 days after inoculation and once a week thereafter, until 8 weeks). These tests were performed in triplicate assay systems and the interpretation of results was carried out from the zone of inhibition of microbial growth (∅ of the halo in mm). For satisfactory susceptibility results (i.e., presence of growth inhibition zone), the determination of MIC against *S. praealtum* extracts can be performed following the guidelines established by the Clinical and Laboratory Standards Institute [39].

### In vitro cytotoxicity assay

In vitro cytotoxicity (CI) of *S. praealtum* extracts was determined by the MTT method (Methylthiazolyldiphenyl-tetrazolium bromide), as previously described [32]. Cells (derived from *Aedes albopictus* mosquito larvae) were poured (1 × 10^4^ cells per well) in microdilution plates with multiple wells (96-well), containing 100 μl of L-15 medium (L-15 Medium [Leibovitz], without L-glutamine, liquid, sterile-filtered, suitable for cell culture. Cat. #L5520, Sigma-Aldrich Co., St. Louis, MO., USA), supplemented with 1% fetal bovine serum (Fetal Bovine Serum, USA origin, sterile-filtered, suitable for cell culture. Cat. #F6178, Sigma-Aldrich Co., St. Louis, MO., USA). Then, microdilution plates were incubated at 37 °C for 72 h under a atmosphere of 5% CO_2_. Shortly after the incubation period, 100 μl aliquots of L-15, added with decreased dilutions of *S. praealtum* extracts (0.039 to 5 mg/ml) were dispensed into the wells of the microdilution plates, which were incubated at room temperature for 48 h. As cellular growth control of *Aedes albopictus*, we used only the culture medium L-15 added with bovine fetal serum. After the reincubation period, aliquots of 10 μl of MTT solution (5 mg/ml) (Thiazolyl Blue Tetrazolium Bromide, powder, BioReagent, suitable for cell culture, suitable for insect cell culture, ≥ 97.5% [HPLC]. Cat. #M5655, Sigma-Aldrich Co., St. Louis, MO., USA) were added to the wells of the microdilution plates and incubated for 4 h at room temperature, to incorporate the MTT cell and create formazan crystals. The reading of results was done by spectrophotometric analysis with wavelength of 600 nm (A_600 nm_). The percentage of in vitro cytotoxicity (CI_%_) was calculated using the following formula: *A* × *B/A* × 100, where *A* and *B* correspond to the values of A_600 nm_ obtained from the control of cell growth (L-15 medium + bovine fetal serum + *Aedes albopictus*) and cell growth in the presence of *S. praealtum* (L-15 medium + bovine fetal serum + *Aedes albopictus* + dilutions of extracts), respectively. In vitro cytotoxicity (CI) of 50% (CI_50_) and 90% (CI_90_) were estimated using linear regression and defined as the extract concentrations capable of reducing 50 and 90%, respectively, of the A_600 nm_ values obtained from cell growth in the presence of *S. praealtum* in comparison with those of the control of cell growth. All these tests were performed in duplicate assay system with control of cell viability.

### In vivo micronucleus assay

Healthy, heterogeneous, young adult male and female *Swiss albinus* (Unib: SW) mice (between 7 and 12 weeks – pubescent period), with a body weight between 30 g and 40 g (i.e., the variation weight between the animals, for each sex, should not exceed ±20% of medium mass) were provided by CEMIB (Multidisciplinary Center for Biological Investigation on Laboratory Animal Science – UNICAMP), and erythrocytes from the bone marrow of these mice were used in the micronucleus assay. Animals were kept in same-sex groups, in polypropylene boxes, in an air-conditioned environment of 22 ± 3 °C, with relative humidity of 50–60 ± 10%, and 12-h day-night cycles (i.e., 12 h light and 12 h dark). Mice were fed with Purina® Labina commercial rations (Nestlé Purina Pet Care Company) and water ad libitum. In addition, they were acclimated to laboratory conditions for 7 days (trial period) before the experiment. At the end of the trial period, each animal was weighed and, according to weight, received 1 ml/100 g body weight of the indicated liquid (negative control, positive control and herbal medicine). After the experimental treatment, animals were euthanized by CO_2_ asphyxiation in adapted acrylic chambers [23, 27]. This research was approved by the Ethics Committee in Research Involving Animals of UNIFENAS (CEPEAU Protocol No. 08A/2014).

Groups of animals (consisting of 5 males and 5 females each) were treated using a single dosing regimen administered by gavage (herbal medicine and negative control) or intraperitoneally (positive control) and two euthanasia times (24 and 48 h), based on the regulatory recommendation regarding the in vivo micronucleus assay: (*i*) Control groups: 150 mM NaCl (negative control), 50 mg/kg of N-Nitroso-N-ethylurea (positive control: NEU, Sigma N8509, CAS No. 759–73-9); (*ii*) Genotoxicity test (herbal medicines): 500, 1000 and 2000 mg/kg of lyophilized extracts of *S. praealtum* leaves diluted in Type 1 water. The maximum tolerated dose (MTD) was defined as the highest dose that can be administered without inducing lethality or excessive toxicity during the study, causing moribund euthanasia, or a dose that produces some indication of toxicity of the bone marrow (e.g. a reduction in the proportion of immature erythrocytes among total erythrocytes in the bone marrow), or 2000 mg/kg [23, 27].

Shortly after euthanasia, the femora were surgically and aseptically removed, and the animals were appropriately discarded. Each femur was sectioned at the proximal end and the contents of the spinal canal were washed with 1.5 ml of a 150 mM NaCl solution and then transferred to a 15 ml centrifuge tube. This material was resuspended with a Pasteur pipette to ensure a random distribution of bone marrow cells. The suspension was then centrifuged at 1000 rpm (*Centrífuga de Bancada Microprocessada*, Mod. NT 810, Nova Técnica Ind. e Com. de Equip. para Laboratório Ltda., Piracicaba, SP, Brasil) for 5 min. The supernatant was discarded and the resulting sediment was resuspended in 500 μl of a 150 mM NaCl solution enhanced with 4% formaldehyde. Slides were smeared (2 slides per animal), then dried at room temperature for 24 h and stained with Leishman’s eosin methylene blue dye (pure dye for 3 min, followed by diluted dye in Type 1 water in a 1:6 proportion for 15 min) to tell apart polychromatic erythrocyte (PCE) from normochromatic erythrocyte (NCE) [23, 27].

Polychromatic erythrocytes (PCEs) were observed at a magnification of 1000× using optical microscopy (Nikon Eclipse E–200), counted (at least 4000 enucleated polychromatic erythrocytes were scored per animal for the incidence of micronucleated polychromatic erythrocytes) with the aid of a digital cell counter (Contador Diferencial CCS02, Kacil Indústria e Comércio Ltda., PE, Brasil) and photographed using an 8.1 Megapixel Digital Camera (DC FWL 150). The number of PCEs and NCEs and the number and frequency of micronucleated polychromatic erythrocytes (MNPCEs) were reported. To evaluate bone marrow toxicity, the ratio of PCE to NCE was also observed. This PCE:NCE ratio indicates the acceleration or inhibition of erythropoiesis and it has been reported to vary with scoring time. A continuous decline in the PCE:NCE ratio may be due to the inhibition of cell division, the killing of erythroblasts, the removal of damaged cells, or dilution of the existing cell pool with newly-formed cells [23, 27].

### Data analysis

Data obtained in the extract disk diffusion testing were submitted to analysis of one-way variance (ANOVA) and medium comparison with Scott-Knott’s test (α = 0.05). IC_50_ and MIC data were used to calculate the selectivity index (SI) of each extract (SI = IC_50_/MIC), as previously reported [40]. Data obtained in the micronucleus assay were submitted to ANOVA using a factorial scheme of 5 × 2 × 2 (treatment × sex × euthanasia time), and medium comparison with Tukey’s test (α = 0.05) using the SAS® version 9.3 computer software [27].

## Results

### Qualitative analysis by GC-MS

**Fig. 1:**
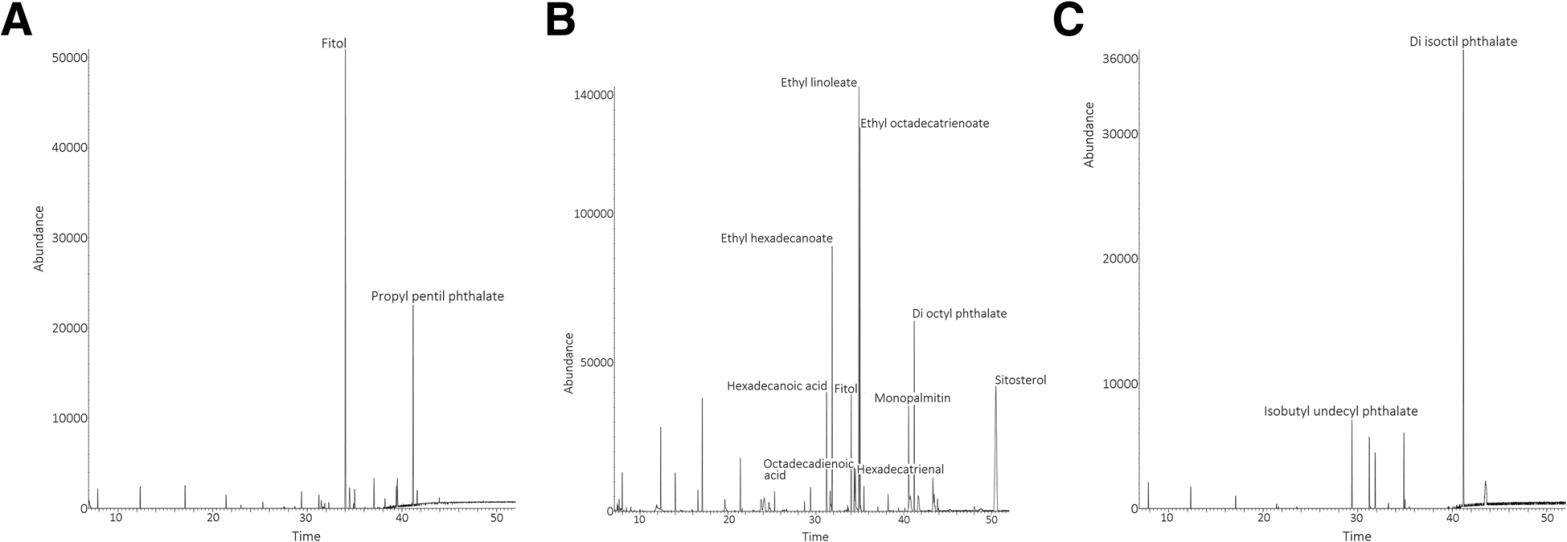
Chromatographic profiles of the lyophilized extracts of leaf (A), stem (B) and root (C) of *S. praealtum* using gas chromatography – mass spectrometry (GC-MS) and mass spectral database (NIST 11)

Chromatographic profiles and mass spectra of lyophilized extracts (stem, leaf and root of *S. praealtum*) were summarized in Table 1 and Fig. 1

**Table 1:** Phytochemical screening (qualitative analysis) of the lyophilized extracts of *S. praealtum* (stem, leaf and root) by GC-MS.

Peak	t_R_	Compound name	%A	Quality
Leaf of *S. praealtum*				
1	34.014	Phytol	60.92	91
2	41.174	Propyl pentil phthalate	30.08	91
Stem of *S. praealtum*				
1	31.218	Hexadecanoic acid	5.56	99
2	31.852	Ethyl hexadecanoate	10.88	96
3	34.017	Phytol	5.06	91
4	34.371	Octadecadienoic acid	2.39	98
5	34.485	Hexadecatrienal	2.95	72
6	34.901	Ethyl linoleate	18.41	99
7	35.016	Ethyl octadecatrienoate	16.81	99
8	40.552	Monopalmitin	4.73	91
9	41.186	Di octyl phthalate	8.62	91
10	50.468	Sitosterol	24.60	99
Root of *S. praealtum*				
1	29.394	Isobutyl undecyl phthalate	11.54	74
2	31.236	Not determined	8.84	–
3	31.850	Not determined	6.07	–
4	34.889	Not determined	7.81	–
5	41.184	Di isoctil phthalate	65.74	91

t_R_: retention time (minutes). %A: percentage of normalized area. Quality indexes > 70 were adopted

### In vitro antimicrobial susceptibility testing and cytotoxicity assay

The in vitro antimicrobial susceptibility profile of gram-negative bacterial strains (*E. coli*, *E. aerogenes*, *S. marcescens*, *P. aeruginosa*, *P. mirabilis* and *S. typhimurium*) against lyophilized extracts of the stem, leaf and root of *S. praealtum*, as determined by the agar diffusion method, showed absence of inhibition zones of microbial growth (∅_mm_ of the halo equivalent to 0). Considering the gram-positive bacterial strains, the presence of the inhibition zone of microbial growth (∅_mm_ of halo) was observed for a greater number of the analyzed strains against the hydroalcoholic extract of the root of *S. praealtum* (4 out of 5 strains: *B. subtilis*, *B. cereus*, *M. luteus* and *S. aureus*), followed by the stem (3 out of 5: *B. cereus*, *M. luteus* and *S. aureus*) and the leaf (2 out of 5: *B. cereus* and *M. luteus*). However, significant statistical differences (*p* < 0.05) were observed only on the inhibition zones of microbial growth (∅_mm_ of halo) formed from the *M. luteus* species and the lyophilized extract of the *S. praealtum* stem. Among the analyzed yeast strains (*C. albicans* and *S. cerevisiae*), only for the *S. cerevisiae* species there were growth inhibition zones against the lyophilized extracts of *S. praealtum* stems and roots. Hydroalcoholic extracts of *S. praealtum* stem, leaf and root did not show any growth inhibition zones against *M. tuberculosis* and *M. bovis* strains. All positive controls (chlorhexidine for bacteria and yeasts; rifamycin to mycobacteria) produced significantly different (*p* < 0.05) growth inhibition zones from those observed in lyophilized extracts of *S. praealtum* stem, leaf and root (Table 2).

**Table 2:** Minimum inhibitory concentration (MIC), minimum microbicidal concentration (MMC), mean values of inhibition halos (M∅_h_ mm) and selectivity index (SI) obtained from in vitro antimicrobial susceptibility testing and cytotoxicity assays of the lyophilized extracts of *S. praealtum*

Microorganism	Lyophilized extract of *S. praealtum* A.DC.												Controls	
	Leaf				Stem				Root				Chlorhexidine	Rifamycin
	MIC/MMC	M∅_h_	IC_50_	SI	MIC/MMC	M∅_h_	IC_50_	SI	MIC/MMC	M∅_h_	IC_50_	SI	M∅_h_	M∅_h_
Gram-negative bacteria														
*E. coli*	25/−	0^A^	4.22	0.17	25/−	0^A^	5.96	0.24	−/−	0^A^	–	–	12.3^D^	*na*
*E. aerogenes*	50/−	0^A^	4.22	0.08	−/−	0^A^	–	–	−/−	0^A^	–	–	9.7^C^	*na*
*S. marcescens*	25/50	0^A^	4.22	0.17	−/−	0^A^	–	–	−/−	0^A^	–	–	11.3^D^	*na*
*P. aeruginosa*	25/−	0^A^	4.22	0.17	−/−	0^A^	–	–	−/−	0^A^	–	–	11.3^D^	*na*
*P. mirabilis*	25/−	0^A^	4.22	0.17	−/−	0^A^	–	–	50/50	0^A^	–	> 1*	9.3^C^	*na*
*S. typhimurium*	25/−	0^A^	4.22	0.17	−/−	0^A^	–	–	−/−	0^A^	–	–	10.3^C^	*na*
Gram-positive bacteria														
*B. subtilis*	50/−	0^A^	4.22	0.08	50/−	0^A^	5.96	0.12	25/25	7.7^C^	–	> 1*	13.7^D^	*na*
*B. cereus*	25/−	7^C^	4.22	0.17	25/−	6.3^C^	5.96	0.24	12.5/−	8.7^C^	–	> 1*	13^D^	*na*
*M. luteus*	12.5/50	7.3^C^	4.22	0.34	25/50	4^B^	5.96	0.24	12.5/50	10.3^C^	–	> 1*	16^E^	*na*
*E. faecalis*	25/−	0^A^	4.22	0.17	25/−	0^A^	5.96	0.24	12.5/−	0^A^	–	> 1*	14.3^D^	*na*
*S. aureus*	25/50	0^A^	4.22	0.17	25/−	6.3^C^	5.96	0.24	12.5/50	8.7^C^	–	> 1*	13.3^D^	*na*
Yeasts														
*C. albicans*	–	0^A^	–	–	–	0^A^	–	–	25/−	0^A^	–	> 1*	13.3^D^	*na*
*S. cerevisiae*	6.25/50	0^A^	4.22	0.68	12.5/50	6.7^C^	5.96	0.90	6.25/50	8.7^C^	–	> 1*	12.7^D^	*na*
Mycobacteria														
*M. tuberculosis*	*na*	0^a^	*na*	*na*	*na*	0^a^	*na*	*na*	*na*	0^A^	*na*	*na*	*na*	11^D^
*M. bovis*	*na*	0^a^	*na*	*na*	*na*	0^a^	*na*	*na*	*na*	0^A^	*na*	*na*	*na*	11^D^

Although without any zone of inhibition of gram-negative bacterial growth, the minimum inhibitory concentration (MIC) revealed (*i*) MIC values equal to 25 mg/ml in five species (*E. coli*, *S. marcescens*, *P. aeruginosa*, *P. mirabilis* and *S. typhimurium*) and 50 mg/ml in one species (*E. aerogenes*) using the lyophilized extract of *S. praealtum* leaves, (*ii*) MIC value equal to 25 mg/ml in one species (*E. coli*) using the *S. praealtum* stem extract, and (*iii*) MIC value equal to 50 mg/ml in one species (*P. mirabilis*) using the *S. praealtum* root extract. For gram-positive bacteria, MIC values were (*i*) equal to 12.5 mg/ml (*M. luteus*), 25 mg/ml (*B. cereus*, *E. faecalis* and *S. aureus*) and 50 mg/ml (*B. subtilis*) using the lyophilized extract of *S. praealtum* leaves, (*ii*) equal to 25 mg/ml (*B. cereus*, *M. luteus*, *E. faecalis* and *S. aureus*) and 50 mg/ml (*B. subtilis*) using the extract of *S. praealtum* stems, (*iii*) and equal to 12.5 mg/ml (*B. cereus*, *M. luteus*, *E. faecalis* and *S. aureus*) and 25 mg/ml (*B. subtilis*) using *S. praealtum* root extracts. Among yeast strains, *S. cerevisiae* showed MIC values equal to 6.25 mg/ml, 12.5 mg/ml and 6.25 mg/ml using lyophilized extracts of *S. praealtum* leaves, stems and roots, respectively. *C. albicans* showed susceptibility only against the *S. praealtum* roots (MIC equal to 25 mg/ml) (Table 2).

Microbicide activities (25–50 mg/ml) of lyophilized extracts of *S. praealtum* were observed only in two species of gram-negative bacteria (*S. marcescens*: leaf; and *P. mirabilis*: root), three species of gram-positive bacteria (*B. subtilis*: root; *M. luteus*: leaf, stem and root; and *S. aureus*: leaf and root) and in the *S. cerevisiae* yeast (leaf, stem and root) (Table 2).

For all gram-negative and gram-positive bacterial species that had a MIC between 12.5 and 50 mg/ml against lyophilized extracts of *S. praealtum* leaves and stems, the SI was < 1.0 (0.08–0.34). A SI < 1 (0.68–0.90) was also observed in the *S. cerevisiae* specie for the lyophilized extracts of *S. praealtum* leaves (MIC equal to 6.25 mg/ml) and stems (MIC equal to 12.5 mg/ml). Since the lyophilized extract of the *S. praealtum* root showed no in vitro cytotoxicity (CI) under tested conditions, the SI of the root extract was considered > 1 against MICs of *P. mirabilis*, *B. subtilis*, *B. cereus*, *M. luteus*, *E. faecalis*, *S. aureus*, *C. albicans* and *S. cerevisiae* (Table 2). These SI were obtained from the results of the percentage of in vitro cytotoxicity (CI_50_ and CI_90_), whose corresponding values ​​from the regression analysis were 4.22 mg/ml (CI_50_) and 8.61 mg/ml (CI_90_) for leaf extracts, 5.96 mg/ml (CI_50_) and 11.14 mg/ml (CI_90_) to stem extracts, and no in vitro cytotoxicity for the *S. praealtum* root extracts (Table 2).

### In vivo micronucleus assay

Given the broad spectrum of antimicrobial action of the lyophilized extract of *S. praealtum* leaves, as noted in this study, only the extracts from leaves have been studied in the in vivo micronucleus assay. Numbers and frequencies of MNPCEs and the PCE/NCE ratio in the mice bone marrow were statistically analyzed for each of the animal groups treated with lyophilized extracts of *S. praealtum* leaves and for each of the groups treated with 150 mM NaCl and N-Nitroso-N-ethylurea (control groups) – genotoxic assay.

For animal groups treated with extracts of *S. praealtum* leaves, analysis of MNPCEs showed no significant differences (*p* < 0.05) between treatment doses (500–2000 mg/kg) and negative control (NaCl). These results suggest an absence of genotoxicity (no clastogenic and/or aneugenic effects) of lyophilized hydroalcoholic extract of *S. praealtum* leaves, regardless of the dose of herbal medicine administration (500–2000 mg/kg) and treatment time (24 and 48 h), but gender-dependent (male and female). The analysis obtained from the PCE/NCE ratio showed significant differences (*p* < 0.05) between the control groups (150 mM NaCl and 50 mg/kg of N-Nitroso-N-ethylurea) and all doses of extracts of *S. praealtum* leaves (500–2000 mg/kg). Therefore, these results suggest that there is a systemic toxicity of the lyophilized hydroalcoholic extract of *S. praealtum* leaves under MN assay conditions, regardless of the herbal medicine dose and the treatment time (24 and 48 h), but gender-dependent (male and female) (Table 3).

**Table 3:** The incidence of MNPCEs and PCE/NCE ratio in bone marrow of male (♂) and female (♀) *Swiss albinus* mice after testing for 24 h and 48 h. Data are from the controls (NaCl and NEU) and an evaluation of the genotoxicity of the lyophilized hydroalcoholic extract of *S. praealtum* leaves

Treatment	MNPCEs (*n*)		MNPCEs (%)		PCE / (PCE + NCE)	
	24 h ^A^	48 h ^A^	24 h ^A^	48 h ^A^	24 h ^A^	48 h ^A^
150 mM NaCl						
♀ ^A (MNPCE); A (PCE/NCE ratio)^	8 ± 3	7 ± 3	0.39 ± 0.09	0.33 ± 0.16	1.00 ± 0.00	1.00 ± 0.00
♂ ^B (MNPCE); B (PCE/NCE ratio)^	8 ± 3	11 ± 1	0.37 ± 0.16	0.53 ± 0.06	1.00 ± 0.00	1.00 ± 0.00
Mean ± SD	8 ± 3 ^A^	9 ± 3 ^A^	0.38 ± 0.12 ^A^	0.43 ± 0.15 ^A^	1.00 ± 0.00 ^A^	1.00 ± 0.00 ^A^
N–Nitroso–N–ethylurea (NEU: 50 mg/kg)						
♀ ^A (MNPCE); A (PCE/NCE ratio)^	27 ± 9	33 ± 3	1.33 ± 0.43	1.63 ± 0.11	0.54 ± 0.04	0.75 ± 0.12
♂ ^B (MNPCE); B (PCE/NCE ratio)^	68 ± 32	35 ± 5	3.33 ± 1.54	1.74 ± 0.27	0.55 ± 0.10	0.54 ± 0.11
Mean ± SD	48 ± 31 ^B^	34 ± 4 ^B^	2.33 ± 1.50 ^B^	1.69 ± 0.20 ^B^	0.54 ± 0.07 ^C^	0.65 ± 0.16 ^C^
*Sedum praealtum* A.DC. (500 mg/kg)						
♀ ^A (MNPCE); A (PCE/NCE ratio)^	9 ± 7	13 ± 8	0.44 ± 0.34	0.60 ± 0.37	0.90 ± 0.04	0.92 ± 0.02
♂ ^B (MNPCE); B (PCE/NCE ratio)^	4 ± 2	5 ± 1	0.21 ± 0.10	0.24 ± 0.03	0.91 ± 0.03	0.87 ± 0.03
Mean ± SD	7 ± 5 ^A^	9 ± 7 ^A^	0.33 ± 0.26 ^A^	0.42 ± 0.31 ^A^	0.90 ± 0.03 ^B^	0.89 ± 0.03 ^B^
*Sedum praealtum* A.DC. (1000 mg/kg)						
♀ ^A (MNPCE); A (PCE/NCE ratio)^	6 ± 1	18 ± 8	0.31 ± 0.07	0.88 ± 0.38	0.91 ± 0.02	0.91 ± 0.01
♂ ^B (MNPCE); B (PCE/NCE ratio)^	6 ± 1	13 ± 1	0.29 ± 0.07	0.63 ± 0.05	0.91 ± 0.02	0.91 ± 0.02
Mean ± SD	6 ± 1 ^A^	15 ± 6 ^A^	0.30 ± 0.07 ^A^	0.76 ± 0.29 ^A^	0.91 ± 0.02 ^B^	0.91 ± 0.01 ^B^
*Sedum praealtum* A.DC. (2000 mg/kg)						
♀ ^A (MNPCE); A (PCE/NCE ratio)^	9 ± 4	9 ± 2	0.46 ± 0.18	0.44 ± 0.09	0.92 ± 0.01	0.91 ± 0.02
♂ ^B (MNPCE); B (PCE/NCE ratio)^	5 ± 4	25 ± 14	0.22 ± 0.18	1.24 ± 0.66	0.90 ± 0.04	0.91 ± 0.04
Mean ± SD	7 ± 4 ^A^	17 ± 13 ^A^	0.34 ± 0.21 ^A^	0.84 ± 0.61 ^A^	0.91 ± 0.03 ^B^	0.91 ± 0.03 ^B^

Means with the same letter (A, B or C) are not significantly different (*p <* 0.05)

## Discussion

Natural products and herbal medicinal plants are a major source of chemical compounds with potential therapeutic applicability [6] and, consequently, these plants can be important healing alternatives. Chemical studies of the *Sedum* species showed several classes of biologically active substances, such as alkaloids, tannins, flavonoids and cyanogenic compounds [1–4]. In addition, the phytochemical characterization of different *Sedum* species and the association between chemical compounds and some biological and pharmacological activities and evolutionary events has been demonstrated [1–4, 17] (Table 4). The phytochemical screening of the lyophilized extracts of *S. praealtum* (stem, leaf and root) by GC-MS reveled the following compounds: leaf of *S. praealtum*: phytol (relative percentage: 60.92%) and propyl pentil phthalate (30.08%); stem of *S. praealtum*: sitosterol (24.6%), ethyl linoleate (18.41%), ethyl octadecatrienoate (16.81%), ethyl hexadecanoate (10.88%), di octyl phthalate (8.62%), hexadecanoic acid (5.56%), phytol (5.06%), monopalmitin (4.73%), hexadecatrienal (2.95%) and octadecadienoic acid (2.39%); and root of *S. praealtum*: di isoctil phthalate (65.74%) and isobutyl undecyl phthalate (11.54%). Of these compounds, phytol [41], hexadecanoic acid [42, 43] and propyl pentil phthalate [44] were potentially associated with certain biological activities. The phytol exert a wide range of biological effects and it is a potential candidate for a broad range of applications in the pharmaceutical and biotechnological industry. Investigations with phytol demonstrated anxiolytic, metabolism-modulating, cytotoxic, antioxidant, autophagy- and apoptosis-inducing, antinociceptive, anti-inflammatory, immune-modulating, and antimicrobial effects [41]. The inhibition of biofilm, extracellular polymeric substances, cell surface hydrophobicity, pyocyanin, pyoveridin and swarming motility of *P. aeruginosa* were observed from the *S. platensis* methanolic extract (major component identified was hexadecanoic acid) [42]. The significant gastroprotective action, antioxidant (DPPH^•^, ABTS^•+^ and FRAP) and anti-*Helicobacter pylori* (MIC of 100 μg/ml) activities were demonstrated from the methanol extract of leaves of *S. involucrata* var. *paniculata* (C. B. Clarke) Munir (twenty-one compounds identified, among them hexadecanoic acid) [43]. Studies involving both compounds di-*n*-propyl phthalate and di-*n*-pentyl phthalate (DPP) showed toxicity to the reproductive system (complete inhibition of fertility or reduced fertility towards both male and female mice) associated with decreased body weight, increased liver weight, decreased testis and epididymis weights, decreased epididymal sperm concentration, and elevated seminiferous tubule atrophy [44].

**Table 4:** Phytochemical characterization studies of *Sedum* spp. and their associations with biological and pharmacological events, and evolutionary and ecological significance

Species	Phytochemical compound	Biological and pharmacological activity
*Sedum tatarinowii* (root, stem and leaves) [17]	Total falconoid (↑mg/g), Polysaccharide (↑mg/g), Free phenol (↓mg/100 g) and Bound phenol (↓mg/100 g).	Antimicrobial test (*E. coli*, *S. aureus*, *S. typhimurium* and *L. monocytogenes*).
*Sedum aizoon* (root, stem and leaves) [17]	Total falconoid (↓mg/g), Polysaccharide (↓mg/g), Free phenol (↑mg/100 g) and Bound phenol (↑mg/100 g).	Antimicrobial test (*E. coli*, *S. aureus*, *S. typhimurium* and *L. monocytogenes*).
*Sedum dendroideum* (leaves) [4]	Flavonoids: kaempferol 3-*O*-α-rhamnopyranosyl-(1 → 2)-β-glucopyranoside-7-*O*-α glucopyranoside; kaempferol 3-*O*-α-rhamnopyranosyl-(1 → 2)-β-glucopyranoside-7-*O*-α-rhamnopyranoside; kaempferol 3-*O*-α-rhamnopyranoside-7-*O*-α-rhamnopyranoside (kaempferitrin); kaempferol 3-*O*-β-glucopyranoside-7-*O*-α-rhamnopyranoside.	In vivo antinociceptive and anti-inflammatory activities (adult male *Swiss* mice).
*Sedum meyeri-johannis* Engler, *S. bourgaei* Hemsley, *S. dendroideum* Moç & Sessé, *S. reptans* Clausen, *S. acre* L., *S. brissemoreti* Hamet, *S. farinosum* Lowe, *S. fusiforme* Lowe, *S. nudum* Aiton, *S. anglicum* Huds., *S. melanantherum* DC., *S. alpestre* Vill., *S. annuum* L., *S. urvillei* DC., *S. litoreum* Guss., *S. stellatum* L., *S. album* L., *S. brevifolium* DC., *S. lydium* Boiss., *S. forsterianum* Sm., *S. montanum* Song. & Perr. spp. *montanum*, *S. rupestre* L. ssp. *erectum* ‘t Hart and *S. sediforme* (Jacq.) Pau. (leaves) [3]	Alkaloids and tannins (proanthocyanidins and esters of gallic acid) in 36 species of the Crassulaceae representing the five largest of Berger’s (1930) six subfamilies, including 23 *Sedum* spp.	Characterization of the alkaloids and tannins (proanthocyanidins and esters of gallic acid) and their evolutionary and ecological significance.
*Sedum telephium ssp. maximum* (leaves) [2]	Flavonol glycosides (kaempferol 3-*O*-β-neohesperidoside-7-*O*-α-rhamnoside and quercetin 3-*O*-β-neohesperidoside-7-*O*-α-rhamnoside), quercetin, kaempferol and their 3-giucosides, 7-rhamnosides and 3,7-dirhamnosides	To investigate the flavonoids constituents.
*Sedum cepaea* (aerial plant material) [1]	Cyanogenic compound (sarmentosin epoxide)	Releases HCN after hydrolysis of the oxiran group to a cyanohydrins.

In particular, *S. praealtum* has been used for a long time in traditional medicine as an analgesic and anti-inflammatory agent because of its beneficial effect on the treatment of eye pain [10], eye swelling [11], ulcer treatment [12, 13], general inflammatory problems [13, 14], as contraception [15], anti-fertilization [16] and antinociceptive activity [13]. However, scientific studies on the *S. praealtum* species aiming to understand its antimicrobial, cytotoxic and genotoxic effects have been scarce so far. Therefore, these observations also drove us to evaluate the antimicrobial (gram-negative and gram-positive bacteria, yeasts and mycobacteria), cytotoxic (*Aedes albopictus* cells) and genotoxic potential (clastogenic and/or aneugenic effects) of the lyophilized hydroalcoholic extract *S. praealtum* using in vitro antimicrobial susceptibility and cytotoxicity tests and the in vivo micronucleus assay.

Scientific evidence showed that the organic extracts (solvents of increasing polarity, e.g., ethanolic and ethyl acetate extracts) of medicinal plants have been considered as best solvents for the extraction of antimicrobial substances when compared with aqueous extracts [32, 45, 46]. Our antimicrobial studies, as determined by the agar diffusion method, showed absence of antimicrobial susceptibility (null growth inhibition zone) of gram-negative bacterial strains (*E. coli*, *E. aerogenes*, *S. marcescens*, *P. aeruginosa*, *P. mirabilis* and *S. typhimurium*) against the lyophilized hydroalcoholic extracts of *S. praealtum* stems, leaves and roots. However, gram-positive bacterial strains showed susceptibility (zone of inhibition of growth) against the root extract (*B. subtilis*, *B. cereus*, *M. luteus* and *S. aureus*), followed by the stem (*B. cereus*, *M. luteus* and *S. aureus*) and leaf (*B. cereus* and *M. luteus*) of *S. praealtum*, with considerable scope of gram-positive bacterial species using root extract, followed by the stem and leaf. In addition, a single smaller growth inhibition zone (*p* < 0.05) was observed on the *M. luteus* specie against the stem extract. The *S. cereviseae* yeast showed susceptibility (zone of inhibition of growth) against the stem and root extracts. The *E. faecalis*, *C. albicans*, *M. tuberculosis* and *M. bovis* strains did not show susceptibility to any parts of the *S. praealtum* extract.

Unlike the agar diffusion method, the broth microdilution showed antimicrobial action of lyophilized extracts of *S. praealtum*, its leaves responsible for the greater spectrum of bacterial species and variable values of MIC against gram-negative bacterial species. These results suggested that (*i*) the lyophilized extracts of *S. praealtum* show gram-negative antibacterial action, showing variable MICs depending on the anatomical part of the plant and the gram-negative bacterial species: leaf (*E. coli*, *S. marcescens*, *P. aeruginosa*, *P. mirabilis* and *S. typhimurium*: MIC = 25 mg/ml; *E. aerogenes*: MIC = 50 mg/ml), stem (*E. coli*: MIC = 25 mg/ml) and root (*P. mirabilis*: MIC = 50 mg/ml); (*ii*) the broth microdilution method has increased sensibility to gram-negative antibacterial tests against the plant extracts of *S. praealtum*, when compared with the agar diffusion method; and (*iii*) bioactive phytochemicals compounds (less polar in nature and/or apolar) of *S. praealtum* with potentially gram-negative antibacterial actions can interact physically and/or chemically with the conditions of the agar diffusion method and, therefore, generate understated data about the antimicrobial susceptibility profile. To the gram-positive bacteria tested, the broth microdilution assay showed antimicrobial action in all lyophilized extracts of *S. praealtum* and variable values of MIC. Thus, these data also reinforce the previous hypothesis about the greater sensitivity of the broth microdilution method, also suggesting that the lyophilized extracts from leaves, stems and roots of *S. praealtum* have gram-positive antibacterial action, showing variable MIC according to the gram-positive bacterial species: leaf (*M. luteus*: 12.5 mg/ml; *B. cereus*, *E. faecalis* and *S. aureus*: 25 mg/ml; *B. subtilis*: 50 mg/ml), stem (*B. cereus*, *M. luteus*, *E. faecalis* and *S. aureus*: 25 mg/ml; *B. subtilis*: 50 mg/ml) and root (*B. cereus*, *M. luteus*, *E. faecalis* and *S. aureus*: 12.5 mg/ml; *B. subtilis*: 25 mg/ml).

The lyophilized extracts of *S. praealtum* leaves and stems did not show any potential of anti-*C. albicans* action, as demonstrated by both methods (i.e. agar diffusion and broth microdilution), except for the extract of *S. praealtum* roots (MIC equal to 25 mg/ml). However, antifungal action of all these extracts was observed against the *S. cerevisiae* yeast assay and associated with more favorable MIC results (leaf and root: 6.25 mg/ml; stem: 12.5 mg/ml). In this case, the broth microdilution method also showed greater sensibility in determining the antifungal action. No in vitro antimycobacterial action was observed using *S. praealtum* extracts, *M. tuberculosis* and *M. bovis* strains, and only the agar diffusion method. Bacteriostatic, bactericidal, fungistatic and fungicidal patterns of *S. praealtum* extracts were observed in the susceptible microbial species, and MMCs values were typically above MIC values, and dependent on or independent of the anatomical parts of *S. praealtum*: e.g., *S. marcescens* (leaf displaying MMC^+^), *P. mirabilis* (root displaying MMC^+^), *B. subtilis* (root displaying MMC^+^), *M. luteus* (leaf, stem and root displaying MMC^+^), *S. aureus* (leaf and root displaying MMC^+^) and *S. cerevisiae* species (leaf, stem and root displaying MMC^+^).

The absence of correlation between agar diffusion and microdilution methods has been observed in some studies on antimicrobial susceptibility and plant extracts [32, 47]. Although both methods have feasible simplicity and relatively low cost, the microdilution method [48] has showed greater sensitivity to results. Several factors can influence the antimicrobial susceptibility analyses employing various phytochemicals compounds (e.g., culture media, pH, oxygen availability, inoculum and incubation conditions) [49] and, consequently, generate irreproducible results among methods (e.g., microbial growth inhibition zone versus MIC). On the other hand, the methods of extraction of phytochemical compounds (e.g., based on the use of organic solvents, which extract compounds with increasing polarity and better diffusion in agar) and interaction, alteration, depletion or destruction of phytochemical compounds, under test conditions, may reflect on the result of antimicrobial action of plant extracts over the micro-organisms [32, 45, 46]. The conditions of plant cultivation are also prevalent with the influence of biotic and abiotic factors, resulting in limiting effects on the production of bioactive metabolites and influencing the antimicrobial effects (e.g., zone of inhibition, MIC and MMC) [50].

This study showed, for the first time in the literature, data on the antimicrobial action of *S. praealtum* for some strains of gram-negative and gram-positive bacteria, yeasts and mycobacteria. A single comparative study on the amount of some active ingredients of *Sedum aizoon* and *Sedum tatarinowii* was conducted to develop and use new sources of plants of Shanxi Huoshan, in China [17]. In this study, the antibacterial assay using the agar diffusion method showed that the polysaccharide extracts of both species of *Sedum* did not have inhibitory effects against *E. coli*, *S. aureus*, *S. typhimurium* and *L. monocytogenes* strains. However, the free phenol extracts had significant inhibitory effects on some bacteria: *S. aizoon* (*S. aureus*, *S. typhimurium* and *L. monocytogenes*) and *S. tatarinowii* (*E. coli*, *S. aureus* and *L. monocytogenes*). Bound phenol or total falconoid extracts of *S. tatarinowii* showed the strongest activity against *L. monocytogenes* (i.e., ∅ of growth inhibition zone). These results provided a theoretical basis for the comprehensive development and utilization of plant resources of Huoshan, in China [17].

In this research, *S. praealtum* extracts were also tested on *Aedes albopictus* cells, to determine the percentage of in vitro cytotoxicity (CI_50_ and CI_90_) and establish a selectivity index (SI=CI_50_/MIC). We found a SI > 1 in the hydroalcoholic extract of the *S. praealtum* root (absence of in vitro cytotoxicity – IC) against the MICs of the *P. mirabilis*, *B. subtilis*, *B. cereus*, *M. luteus*, *E. faecalis*, *S. aureus*, *C. albicans* and *S. cerevisiae* species. However, such a suggestion could be confirmed from the others in vitro cytotoxicity tests (e.g., human keratinocytes – HaCaT) and using a higher concentration range (e.g., ≤200 mg/ml and ≥ 5 mg/ml). These results suggested the presence of potential cytotoxic phytochemical compounds in *S. praealtum* leaves and stems, contributing to the SI < 1 values. MIC and IC_50_ data have been used to calculate the selectivity index (SI) of (phyto)chemical compounds as an estimate of a therapeutic window and a mechanism to identify candidates for effectiveness studies in mice [40]. An SI ≥ 1 can be considered acceptable [32, 40].

Genotoxicity studies of *S. praealtum* have not been observed in the literature so far, being presented for the first time in this research. The in vivo micronucleus assay was performed with lyophilized extracts of *S. praealtum* leaves, given their broad spectrum of antimicrobial action (gram-negative and gram-positive bacteria and yeast). Data showed no statistically significant differences between the experimental treatments and the negative control of the assay, suggesting the absence of genotoxicity (clastogenic and/or aneugenic) of the hydroalcoholic extract of *S. praealtum* leaves, regardless of herbal medicine doses (0.5–2 g/kg) and treatment times (24 h: acute effect; 48 h: chronic effect), but depending on the sex of the mouse (male and female). The PCE/NCE ratio was analyzed as a measure of toxicity in the bone marrow (systemic toxicity). This PCE/NCE ratio is considered an indicator of accelerating or inhibiting erythropoiesis and has been reported to vary with time. A continuous decline in the PCE/NCE ratio may be due to the inhibition of cell division, the death of erythroblasts, the removal of damaged cells, or the dilution of the total of existing cells with the newly-formed cells [23, 27]. This analysis revealed statistically significant differences between the control groups of animals (150 mM NaCl; 50 mg/kg NEU) and all experimental treatments (i.e., intermediate PCE/NCE ratio to those of the negative and positive controls). Under in vivo micronucleus assay conditions, these data suggested bone marrow toxicity (i.e., systemic toxicity) promoted by the extract of *S. praealtum* leaves, regardless of herbal medicine doses (0.5–2 g/kg) and of treatment times (24 and 48 h), but gender-dependent (male and female mouse).

The results of toxicity in mice bone marrow (systemic toxicity) corroborate with the observations about the in vitro cytotoxicity (*Aedes albopictus* cells), as well as about the harmful physiological (contraceptive action) [15, 16] and cellular modulations (inhibition of sperm motility) [16] previously reported for the *S. praealtum* species. In addition, the acute toxicity of the lyophilized aqueous extract of the aerial parts of *S. praealtum* was evaluated in experiments carried out in adult female Wistar rats. In five groups of four rats, the lethality was assessed using death within 7 days as an index of toxicity following PO administration of lyophilized extract (from 500 to 3000 mg/kg body weight), and no deaths were registered [14]. This toxicological result of *S. praealtum* did not show toxic effects at evaluated doses in comparison with the micronucleus assay (i.e., acute and chronic systemic toxicity), and this could be explained, at least in part, by differences between methods of extraction of phytochemical compounds (i.e., hydroalcoholic versus aqueous).

## Conclusions

This was the first scientific study on the in vivo genotoxic and in vitro antimicrobial and cytotoxic potential of *S. praealtum* (Balsam). The extract of *S. praealtum* leaves showed antimicrobial action (static and/or microbicide) of broad spectrum and variable MICs for gram-negative (*E. aerogenes*, *E. coli*, *P. aeruginosa*, *P. mirabilis*, *S. marcescens* and *S. typhimurium*), gram-positive (*B. cereus*, *B. subtilis*, *E. faecalis*, *M. luteus* and *S. aureus*) bacteria and only one yeast species (*S. cerevisiae*), unacceptable for clinical applications of this nature. Stem (SI < 1) and root (SI > 1) extracts showed more restricted antimicrobial effects (i.e., gram-positive bacteria, *E. coli* and *S. cerevisiae* [stem]; gram-positive bacteria, *P. mirabilis*, *C. albicans* and *S. cerevisiae* [root]). Although data on in vitro cytotoxicity and root SI under testing conditions were favorable, new assays could confirm its missing toxicity. Antimycobacterial action (*M. tuberculosis* and *M. bovis*) was not observed. The lyophilized extracts of *S. praealtum* leaves showed non-genotoxic effects (i.e., absence of clastogenic and/or aneugenic mechanisms) and systemic toxicity (i.e., toxicity in mice bone marrow), regardless of herbal medicine doses (0.5–2 g/kg) and treatment times (24 h: acute effect; 48 h: chronic effect), but gender-dependent (male and female).

Partly, the sum of these results provides a foundation for a comprehensive use and development of plant resources, especially *S. praealtum*. However, the advanced phytochemical characterization together with pharmacological and pharmacogenomics studies (e.g., *Salmonella typhimurium* test as an indicator of potential carcinogenicity for mammals [Ames test]; gene mutation test in mammalian cells [mouse lymphoma assay]; in vitro aneuploidy and cytogenetic tests; in vitro micronucleus test in cultured cells; fluorescent in situ hybridization [FISH] test for mutagenesis; comet test to detect DNA damage and repair in individual cells; functional genomic and proteomic tests for mutagenesis [cDNA microarrays and other array analyses]; DNA nicking assay) of *S. praealtum* extracts and oils should be conducted to characterize their potential effects and action mechanisms, in addition to establishing limits for human consumption, outlining the potential health risks, and implement rational strategies and chemo-preventive measures.

## Data Availability

The datasets used and/or analysed during the current study are available from the corresponding author on reasonable request.

## Abbreviations

BHI: Brain heart infusion
CFU: Colony forming units
CI: Cytotoxicity index
IC_50_: Cytotoxicity index of 50% – concentrations capable of reducing 50%
IC_90_: Cytotoxicity index of 90% – concentrations capable of reducing 90%
HaCaT: Human keratinocytes
MTD: Maximum tolerated dose
MTT: Methylthiazolyldiphenyl-tetrazolium bromide
MNPCEs: Micronucleated polychromatic erythrocytes
MIC: Minimum inhibitory concentration
MMC: Minimum microbicide concentration
NEU: N-nitroso-N-ethylurea
NCEs: Normochromatic erythrocytes
PO administration: Oral administration: from “per os”, the Latin for “by mouth”
PCEs: Polychromatic erythrocytes
SDA: Sabouraud dextrose agar
SI: Selectivity index

## References

[1] Nahrstedt A, Walther A, Wray V. Sarmentosin epoxide, a new cyanogenic compound from *Sedum cepaea*. Phytochemistry. 1982;21:107–110. https://doi.org/10.1016/0031-9422(82)80023-X.

[2] Mulinacci N, Vincieri FF, Baldi A, Bambagiotti-Alberti M, Sendl A, Wagner H. Flavonol glycosides from *Sedum telephium* subspecies maximum leaves. Phytochemistry. 1995;38:531–533. https://doi.org/10.1016/0031-9422(94)00554-7.

[3] Stevens JF, Hart HT, Roeland CHJ, Ham V, Elema ET, Van den Ent MMVX, Wildeboer M, Zwaving JH. Distribution of alkaloids and tannins in the Crassulaceae. Biochem Syst Ecol 1995;23(2):157–165. https://doi.org/10.1016/0305-1978(95)00082-6.

[4] Melo GO, Malvar DC, Vanderlinde FA, Pires PA, Côrtes WS, Filho PG, Muzitano MF, Kaiser CR, Costa SS. Phytochemical and pharmacological study of *Sedum dendroideum* leaf juice. J Ethnopharmacol 2005:102;217–220. https://doi.org/10.1016/j.jep.2005.06.015. 10.1016/j.jep.2005.06.015 16054793

[5] Clausen RT (1959). Sedum of the trans-Mexican Volcanic Belt: an exposition of taxonomic methods.

[6] Rzedowski J, Rzedowski GC (1985). Flora fanerogámica del Valle de México.

[7] Kerguélen M (1999). Index synonymique de la Flore de France.

[8] Lino PL, Voll CE, Heiffig LS, Aguila JS, Dias CTS, Minami K (2008). Produção de mudas de balsamo (*Sedum dendroideum* subsp. *Praealtum* (DC.) R.T. Clausen). Hortic Bras.

[9] Lorenzi H, Souza HM (2001). Plantas Ornamentais no Brasil – Arbustivas, Herbáceas e Trepadeiras.

[10] Cruz M (1964). Libellus de medicinalibus indorum herbis: Aztec manuscript (1552).

[11] Martínez M (1967). Las Plantas Medicinales de México.

[12] Carlini EA, Palermo Neto J, Almeida ET, Marigo C (1970). Úlcera por contenção em ratos: ação protetora de extrato aquoso de bálsamo. Estudo Preliminar An Acad Bras Ciênc.

[13] Melo GO, Malvar DC, Vanderlinde FA, Rocha FF, Pires PA, Costa EA, de Matos LG, Kaiser CR, Costa SS. Antinociceptive and anti-inflammatory kaempferol glycosides from *Sedum dendroideum*. J Ethnopharmacol 2009;124:228–232. https://doi.org/10.1016/j.jep.2009.04.024. 10.1016/j.jep.2009.04.024 19397977

[14] Meléndez Camargo ME, Buendía Romero M, Ramos Zamora D, Cardona Carrillo P, Villarreal Maldonado ME (2002). Study of the anti-inflammatory effect of *Sedum praealtum* (Siempreviva) in the rat: dose-dependent response. Proc West Pharmacol Soc.

[15] Silva-Torres R, Montellano-Rosales H, Ramos-Zamora D, Castro-Mussot ME, Cerda-García-Rojas CM (2003). Spermicidal activity of the crude ethanol extract of *Sedum praealtum* in mice. J Ethnopharmacol.

[16] García Pineda J, Wens MA, Valencia A, Gallegos A (1986). Acción de *Sedum dendroideum* sobre la actividad funcional de los espermatozoides. Archivos de investigación médica.

[17] Hu QP, Xu JG (2012). Active components and antimicrobial activity in two *Sedum* species grown in Shanxi Huoshan. China J Food Agric Environ.

[18] Sikkema J, de Bont JA, Poolman B (1995). Mechanisms of membrane toxicity of hydrocarbons. Microbiol Rev.

[19] Ames BN. Dietary carcinogens and anticarcinogens. Oxygen radicals and degenerative diseases. Science. 1983;221:1256–1264. https://doi.org/10.1126/science.6351251. 10.1126/science.6351251 6351251

[20] Purves D, Harvey C, Tweats D, Lumley CE. Genotoxicity testing: current practices and strategies used by the pharmaceutical industry. Mutagenesis. 1995;10:297–312. https://doi.org/10.1093/mutage/10.4.297. 10.1093/mutage/10.4.297 7476265

[21] Varanda EA (2006). Atividade mutagênica de plantas medicinais. Rev Ciênc Farm Básica Apl.

[22] Mateuca R, Lombaert N, Aka PV, Decordier I, Kirsch-Volders M (2006). Chromosomal changes: induction, detection methods and applicability in human biomonitoring. Biochimie..

[23] Organisation for Economic Co-operation and Development. Guideline for the Testing of Chemicals: Mammalian Erythrocyte Micronucleus Test. Paris, Guideline 474; 2016.

[24] Indart A, Viana M, Clapés S, Izquierdo L, Bonet B. Clastogenic and cytotoxic effects of lipid peroxidation products generated in culinary oils submitted to thermal stress. Food Chem Toxicol 2007;45:1963–1967. https://doi.org/10.1016/j.fct.2007.04.019. 10.1016/j.fct.2007.04.019 17573172

[25] Chandrasekaran CV, Sundarajan K, Gupta A, Srikanth HS, Edwin J, Agarwal A (2001). Evaluation of the genotoxic potential of standardized extract of *Glycyrrhiza glabra* (GutGard™). Regul Toxicol Pharmacol.

[26] Alves JM, Munari CC, Monteiro Neto MAB, Furtado RA, Senedese JM, Bastos JK, Tavares DC (2013). *In vivo* protective effect of *Copaifera langsdorffii* hydroalcoholic extract on micronuclei induction by doxorubicin. J Appl Toxicol.

[27] Boriollo MF, Souza LS, Resende MR, Silva TA, Oliveira NM, Resck MC, Dias CT, Fiorini JE. Nongenotoxic effects and a reduction of the DXR-induced genotoxic effects of *Helianthus annuus* Linné (sunflower) seeds revealed by micronucleus assays in mouse bone marrow. BMC Complement Altern Med 2014;2:121. https://doi.org/10.1186/1472-6882-14-121. 10.1186/1472-6882-14-121 PMC3992159 24694203

[28] Porto, RS: *Conyza canadensis*: determination of bioactive compounds and evaluation of antifungal activity. (2015) http://repositorio.unicamp.br/handle/REPOSIP/249439 . Accessed 18 Dec 2019].

[29] CLSI (2009). Method for antifungal disk diffusion susceptibility testing of yeasts: approved guideline—second edition. CLSI document M44-A2.

[30] CLSI (2012). Performance standards for antimicrobial disk susceptibility tests; approved standard—eleventh edition. CLSI document M02-A11.

[31] CLSI (2012). Performance standards for antimicrobial susceptibility tests; twenty-second informational supplement. CLSI document M100-S22.

[32] Silva JJ, Cerdeira CD, Chavasco JM, Cintra AB, Silva CB, Mendonça AN, Ishikawa T, Boriollo MF, Chavasco JK. *In vitro* screening antibacterial activity of *Bidens pilosa* Linné and *Annona crassiflora* Mart. Against oxacillin resistant *Staphylococcus aureus* (ORSA) from the aerial environment at the dental clinic. Rev Inst Med Trop São Paulo 2014;56:333–340. https://doi.org/10.1590/S0036-46652014000400011. 10.1590/S0036-46652014000400011 PMC4131820 25076435

[33] CLSI (2008). Reference method for broth dilution antifungal susceptibility testing of yeasts: approved standard—third edition. CLSI document M27-A3.

[34] CLSI (2012). Methods for dilution antimicrobial susceptibility tests f or Bacteria that grow aerobically; approved standard—ninth edition. CLSI document M07-A9.

[35] CLSI (2012). Reference method for broth dilution antifungal susceptibility testing of yeasts; fourth informational supplement. CLSI document M27-S4.

[36] Martin A, Camacho M, Portaels F, Palomino JC. Resazurin microtiter assay plate testing of *Mycobacterium tuberculosis* susceptibilities to second-line drugs: rapid, simple, and inexpensive method. Antimicrob Agents Chemother 2003;47:3616–3619. https://doi.org/10.1128/AAC.47.11.3616-3619.2003. 10.1128/AAC.47.11.3616-3619.2003 PMC253784 14576129

[37] Cantón E, Pemán J, Viudes A, Quindós G, Gobernado M, Espinel-Ingroff A. Minimum fungicidal concentrations of amphotericin B for bloodstream *Candida* species. Diagn Microbiol Infect Dis 2003;45:203–206. https://doi.org/10.1016/S0732-8893(02)00525-4. 10.1016/s0732-8893(02)00525-4 12663162

[38] Moore CB, Walls CM, Denning DW. *In vitro* activities of terbinafine against *Aspergillus* species in comparison with those of itraconazole and amphotericin B. Antimicrob Agents Chemother 2001;45:1882–1885. https://doi.org/10.1128/AAC.45.6.1882-1885.2001. 10.1128/AAC.45.6.1882-1885.2001 PMC90563 11353643

[39] CLSI (2011). Susceptibility testing of mycobacteria, Nocardiae, and other aerobic Actinomycetes; approved second—edition. CLSI document M24-A2.

[40] Protopopova M, Hanrahan C, Nikonenko B, Samala R, Chen P, Gearhart J, Einck L, Nacy CA. Identification of a new antitubercular drug candidate, SQ109, from a combinatorial library of 1,2-ethylenediamines. J Antimicrob Chemother 2005;56:968–974. https://doi.org/10.1093/jac/dki319. 10.1093/jac/dki319 16172107

[41] Islam MT, Ali ES, Uddin SJ, Shaw S, Islam MA, Ahmed MI, Chandra Shill M, Karmakar UK, Yarla NS, Khan IN, Billah MM, Pieczynska MD, Zengin G, Malainer C, Nicoletti F, Gulei D, Berindan-Neagoe I, Apostolov A, Banach M, Yeung AWK, El-Demerdash A, Xiao J, Dey P, Yele S, Jóźwik A, Strzałkowska N, Marchewka J, Rengasamy KRR, Horbańczuk J, Kamal MA, Mubarak MS, Mishra SK, Shilpi JA, Atanasov AG. Phytol: A review of biomedical activities. Food Chem Toxicol. 2018;121:82–94. https://doi.org/10.1016/j.fct.2018.08.032. 10.1016/j.fct.2018.08.032 30130593

[42] LewisOscar F, Nithya C, Alharbi SA, Alharbi NS, Thajuddin N. *In vitro* and *in silico* attenuation of quorum sensing mediated pathogenicity in *Pseudomonas aeruginosa* using *Spirulina platensis*. Microb Pathog 2018;116:246–256. https://doi.org/10.1016/j.micpath.2018.01.046. 10.1016/j.micpath.2018.01.046 29409746

[43] Sreeja PS, Arunachalam K, Saikumar S, Kasipandi M, Dhivya S, Murugan R, Parimelazhagan T. Gastroprotective effect and mode of action of methanol extract of *Sphenodesme involucrata* var. *paniculata* (C.B. Clarke) Munir (Lamiaceae) leaves on experimental gastric ulcer models. Biomed Pharmacother 2018;97:1109–1118. https://doi.org/10.1016/j.biopha.2017.11.030. 10.1016/j.biopha.2017.11.030 29136948

[44] Heindel JJ, Gulati DK, Mounce RC, Russell SR, Lamb 4^th^ JC. Reproductive toxicity of three phthalic acid esters in a continuous breeding protocol Fundam Appl Toxicol 1989;12:508–518. https://doi.org/10.1016/0272-0590(89)90024-9. 10.1016/0272-0590(89)90024-9 2731665

[45] Rojas JJ, Ochoa VJ, Ocampo SA, Muñoz JF. Screening for antimicrobial activity of ten medicinal plants used in Colombian folkloric medicine: a possible alternative in the treatment of non-nosocomial infections. BMC Complement Altern Med 2006;17:2. https://doi.org/10.1186/1472-6882-6-2. 10.1186/1472-6882-6-2 PMC1395329 16483385

[46] Mattana CM, Satorres SE, Sosa A, Fusco M, Alcará LE. Antibacterial activity of extracts of acacia aroma against methicillin-resistant and methicillin-sensitive *Staphylococcus*. Braz J Microbiol 2010;41:581–587. https://doi.org/10.1590/S1517-83822010000300007. 10.1590/S1517-83822010000300007 PMC3768637 24031532

[47] Chavasco JM, Prado E Feliphe BHM, Cerdeira CD, Leandro FD, Coelho LFL, Silva JJ, Chavasco JK, Dias ALT. Evaluation of antimicrobial and cytotoxic activities of plant extracts from southern Minas Gerais cerrado. Rev Inst Med Trop São Paulo. 2014;56:13–20. http://dx.doi.org/10.1590/S0036-46652014000100002. 10.1590/S0036-46652014000100002 PMC4085825 24553603

[48] Eloff JN. A sensitive and quick microplate method to determine the minimal inhibitory concentration of plant extracts for bacteria. Planta Med 1998;64:711–713. http://dx.doi.org/10.1055/s-2006-957563. 10.1055/s-2006-957563 9933989

[49] Ostrosky EA, Mizumoto MK, Lima MEL, Kaneko TM, Nishikawa SO, Freitas BR. Methods for evaluation of the antimicrobial activity and determination of minimum inhibitory concentration (MIC) of plant extracts. Rev Bras Farmacogn 2008;18:301–307. http://dx.doi.org/10.1590/S0102-695X2008000200026.

[50] Gobbo-Neto L, Lopes NP. Plantas medicinais: fatores de influência no conteúdo de metabólitos secundários. Quím Nova 2007;30:374–381. http://dx.doi.org/10.1590/S0100-40422007000200026.

